# Failed anterior cruciate ligament reconstructions have both increased posterior tibial slope and increased posterior tibial plateau offset

**DOI:** 10.1002/jeo2.70620

**Published:** 2026-01-12

**Authors:** Romed P. Vieider, Robert E. Bilodeau, Mahmut E. Kayaalp, Tyler M. Hauer, Karina Dias, Ting Cong, Jonathan D. Hughes, Volker Musahl

**Affiliations:** ^1^ Department of Orthopaedic Surgery, UPMC Freddie Fu Sports Medicine Center University of Pittsburgh Pittsburgh Pittsburgh USA; ^2^ Department of Sports Orthopaedics, TUM University Hospital Technical University of Munich Munich Germany; ^3^ Department of Orthopaedics and Traumatology, Istanbul Fatih Sultan Mehmet Training and Research Hospital University of Health Sciences Istanbul Türkiye; ^4^ Division of Orthopaedic Surgery, University Health Network University of Toronto Toronto Ontario Canada

**Keywords:** ACL, bony morphology, posterior tibial plateau offset, posterior tibial slope, slope reducing osteotomy

## Abstract

**Purpose:**

This study introduces the posterior tibial plateau offset (PTO). It was hypothesised that (1) the PTO is reliably quantifiable and (2) correlates with the posterior tibial slope (PTS).

**Methods:**

The study involved lateral radiographs of patients who sustained an anterior cruciate ligament (ACL) graft failure and a control group. Exclusion criteria were skeletal immaturity, osteoarthritis (Kellgren and Lawrence grade > I), lateral radiographs with <15 cm of tibial shaft, or malrotated radiographs. The PTS was measured in both groups. The PTO was defined as the relative distance from the posterior tibial plateau to the tibial shaft axis in relation to the sagittal diameter of the tibial plateau (in%). Linear correlation assessed the PTS to PTO association.

**Results:**

A total of 146 patients (ACL graft failure, *n* = 103; control group, *n* = 43; 45% female, 60% left knees) were included. Mean overall PTO was 82% ± 7% (1%–35%), and mean PTS was 12° ± 3 (7–20). The PTS and PTO showed a moderate positive correlation in the overall collective (*r* = 0.5; *p* < 0.001), ACL graft failure group (*r* = 0.49; *p* < 0.001), and control group (*r* = 0.69; *p* < 0.001), indicating a higher slope corresponded to a greater PTO. There was no statistical difference in PTO between the ACL failure and the control group (82% vs. 83%, *p* < 0.05). ICC between three raters was (0.8–0.9; *p* < 0.05).

**Conclusion:**

The PTO is a simple and repeatable measurement. A higher PTS is associated with a greater PTO, and the variability of the PTO across the patients with ACL graft failure and the control group was high. When planning osteotomy levels in the highly sloped proximal tibia, the PTO may be considered to optimise individualised planning for patients with failed ACL reconstruction.

**Level of Evidence:**

Level V.

**Abbreviations**
ACL: anterior cruciate ligament
HTO: high tibial osteotomy
mm: millimetres
MRI: magnetic resonance imaging
PTO: posterior tibial plateau offset
PTS: posterior tibial slope

## INTRODUCTION

A thorough understanding of the proximal tibial morphology is essential in knee‐preserving surgery [14, 15, 17, 25]. Several parameters have been described to quantify morphological characteristics of the proximal tibia [5, 6, 12, 13]. In the sagittal plane, the most frequently discussed parameter is the posterior tibial slope (PTS). The PTS quantifies the posterior inclination of the tibial plateau in relation to its shaft and is most frequently measured on a true lateral radiograph [10, 19, 24].

The PTS does not characterise the anteroposterior orientation of the tibial plateau related to the tibial shaft in the sagittal plane. The authors define this position as the posterior tibial plateau offset and aimed to propose a parameter to quantify the posterior tibial plateau offset (PTO) on the lateral radiograph. The variation in PTO may have important clinical implications. For example, differences in PTO could affect the required length of osteotomy cuts when planning an anterior closing wedge high tibial osteotomy (HTO). In addition, PTO variation may influence knee biomechanics and kinematics.

Therefore, this study aims to quantify the PTO on lateral radiographs. The purpose of this study was to define a repeatable measurement method to quantify the PTO. The study's hypothesis was (1) The PTO is reliably quantifiable, and it positively correlates with the PTS. (2) There is a high interindividual variability in PTO values.

## METHODS

**Figure 1:**
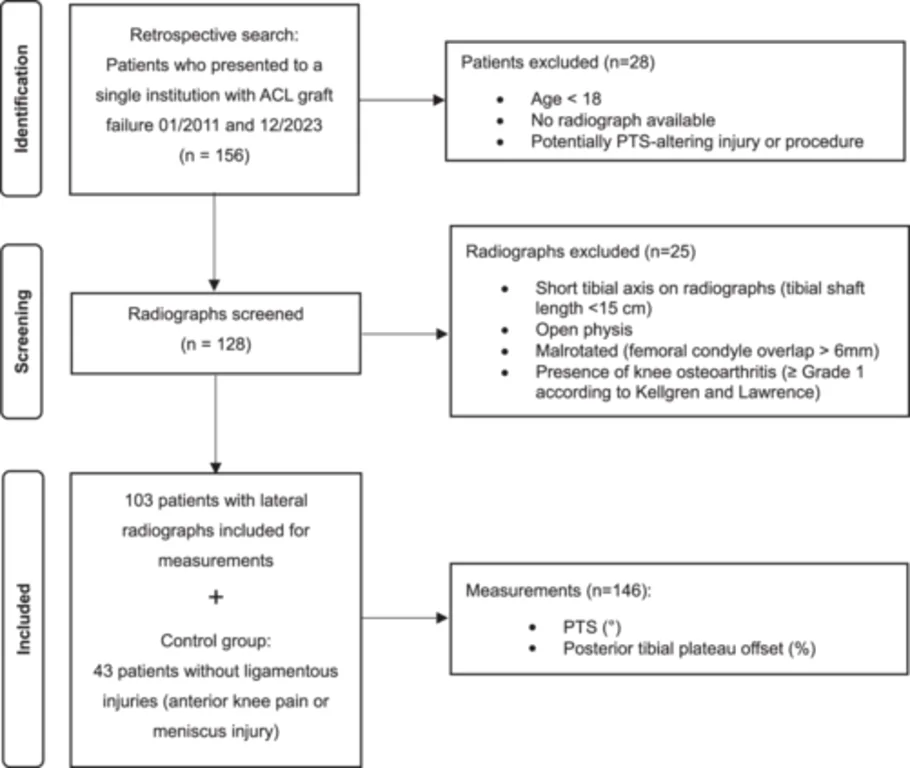
Flowchart of patient selection for the measurement of medial posterior tibial slope (PTS) and posterior tibial plateau offset in patients with anterior cruciate ligament (ACL) graft failure and the control group.

Lateral knee radiographs of all patients >18 years of age who presented with an anterior cruciate ligament (ACL) graft failure between 01/2022 and 12/2023 were included in the study. A control group was established for individuals who underwent lateral radiographs due to anterior knee pain or meniscus tears, but without ligamentous injuries, to account for confounding factors in the ACL graft failure group other than the PTS. Lateral knee radiographs with a short tibial shaft axis (tibial shaft length < 15 cm), malrotated radiographs (defined as a posterior femoral condyle overlap > 6 mm [26]), and radiographs from patients with knee osteoarthritis. Grade I or higher according to Kellgren and Lawrence [16], or a history of potentially PTS‐altering injuries or procedures, were excluded (Figure 1). Demographic data (age, sex and side) of the patients were analysed. All measurements were performed using the picture archive and communication system (PACS, Koninklijke Philips N.V.).

**Figure 2:**
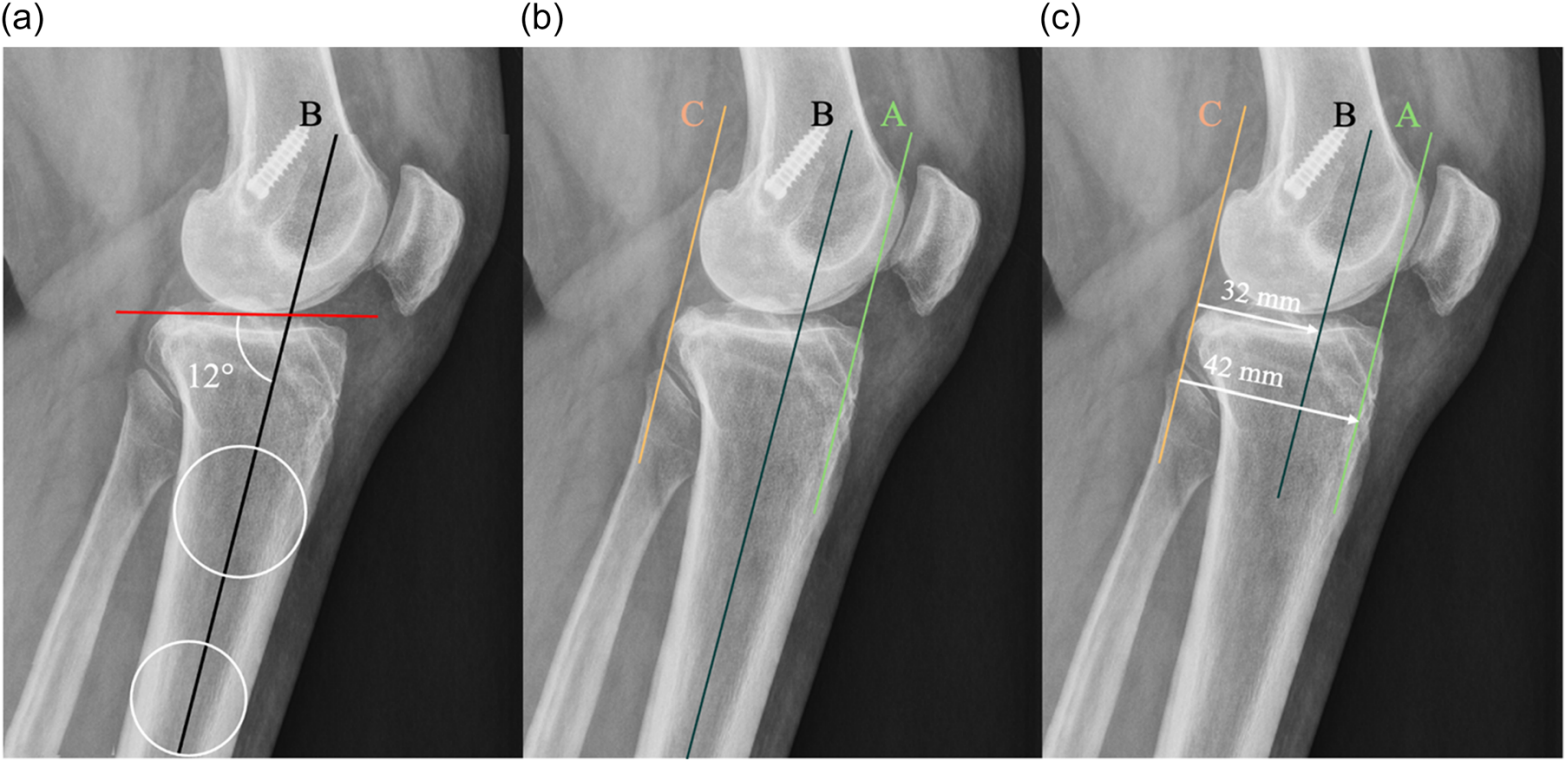
True lateral radiograph of a left knee with high posterior tibial offset of 74%. (a) The medial posterior tibial slope was defined by the angle between the anatomical tibial shaft axis (B, black line) and a line tangent to the medial tibial plateau, measuring 12° in the current example. (b) The posterior tibia plateau offset was then determined as follows: First, the anterior (A, green line) and posterior (C, orange line) tibial articular margins of the plateau were identified, and parallel tangents to the tibial axis were placed. The margins (A and C) were located at the most anterior and posterior edges of the tibial joint surface. The tangent points were defined at the locations where the tibial joint surface begins to “fall off,” and the tangent lines were positioned accordingly. (c) The posterior tibial plateau offset was then determined by the distance between C and B (in image 1c: 32 mm) divided by the distance from C and A (in image 1c: 42 mm), which resulted in 0.74, meaning the anatomical axis intersects the tibial plateau at 26% from an anteroposterior perspective.

For each radiograph, the PTS was measured according to the most frequently used method (Figure 2a) [8, 23]. To determine the anatomical tibial shaft axis, two circles were placed at points 5 and 15 cm distal to the tibial joint surface, aligned with the anterior and posterior cortices. The midpoint of each circle was identified, and a line connecting these midpoints was defined as the anatomical tibial shaft axis. The PTS was defined as the angle between a tangent of the medial tibial plateau and a perpendicular line of the proximal tibial shaft axis (Figure 2a) [8].

To assess the posterior offset of the tibial plateau, two lines parallel to the tibial shaft axis were drawn. The first line was positioned tangentially at the most anterior margin of the medial tibial plateau, while the second was placed at the most posterior margin of the medial tibial plateau. The distance between these two tangential lines and the distance from the tibial shaft axis to the posterior tangential line was measured in millimetres (Figure 2b).

To provide a normalised parameter for the tibial shaft axis position in relation to the tangential reference lines, the percentage was calculated by dividing the distance from the tibial shaft axis to the posterior tangential line by the total distance between the two tangential lines. This percentage was subtracted from 100 to enable standardised comparisons of the tibial shaft axis intersecting the tibial plateau across individuals (Figure 2c).

### Statistical analysis

Descriptive statistics and tests for significance were calculated using SPSS 27.0 (IBM‐SPSS) and displayed by graphs and tables. After testing the data homogeneity of variance (Levene test) and normal distribution (Shapiro–Wilk test), continuous variables were presented as mean and standard deviation (normally distributed) or median and 25%–75% interquartile range (non‐normally distributed). A paired t‐test (normally distributed data) or Wilcoxon rank sum test (non‐normally distributed data) was used for group comparison of continuous variables. Due to the predefined ACL graft failure group, a power analysis was conducted for the control group to determine the necessary sample size for pairwise comparisons of the posterior tibial plateau offset measurement. Based on 10 preliminary measurements (PTO and PTS) in a sample of the control group, a mean effect size of *d* = 0.4 was detected with a two‐sided significance at *α* = 0.05. Achieving an adequate power (1‐β = 0.8) necessitated a sample size (*n*) of 43 radiographs, as determined by the analysis. A Pearson correlation was used to analyse the correlation between the PTS and posterior tibial plateau offset. The calculation utilised G*Power 3.1.9.6 software [8].

To determine the intrareader reliability PTS and posterior tibial offset measurements, a single rater (R.P.V.) performed the measurements at a time interval of 3 months between measurements, and the Pearson correlation coefficient was calculated. The intraclass correlation coefficient (ICC) of three raters (R.V., R.B. and T.H.) was calculated to assess the interrater reliability of the measurements and interpreted according to Koo and Li (<0.50 poor, 0.50–0.75 moderate, 0.75–0.90 good, >0.90 excellent) [17].

## RESULTS

**Figure 3:**
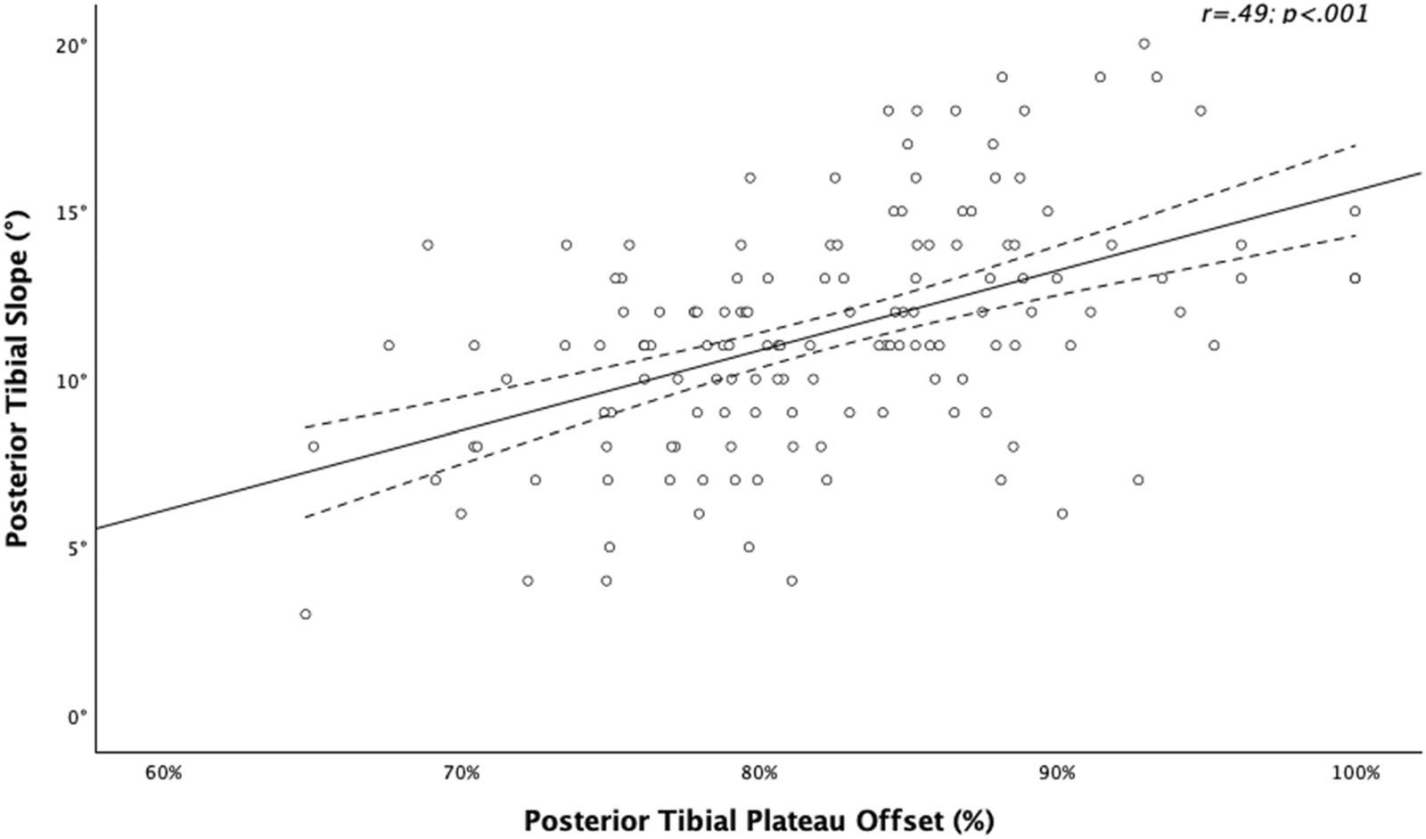
Scatter plot with a trendline (black line) and confidence intervals (dotted lines) illustrating the positive correlation of the posterior tibial plateau offset (%) and the posterior tibial slope (°).

**Figure 4:**
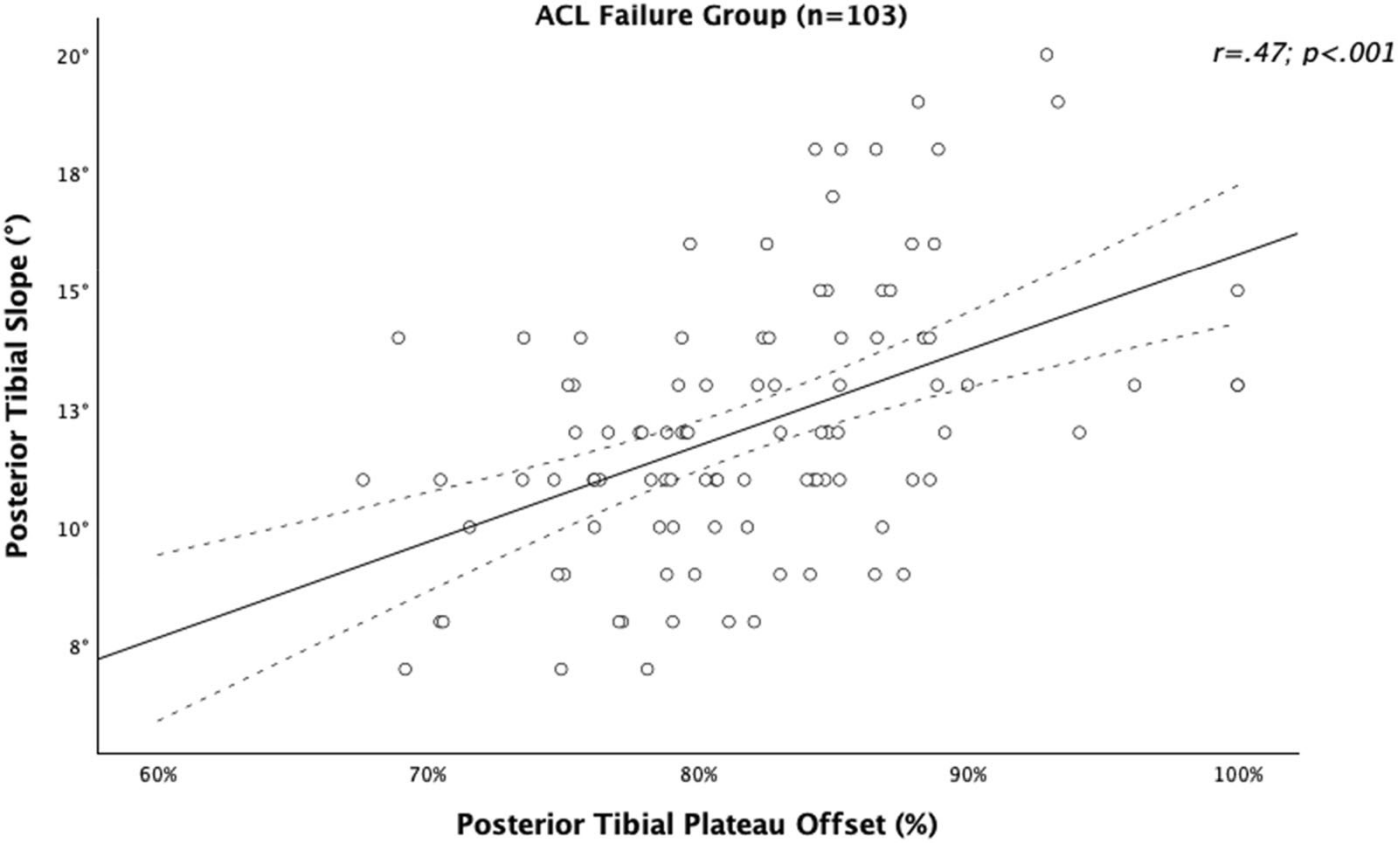
Scatter plot with a trendline (black line) and confidence intervals (dotted lines) illustrating the positive correlation of the posterior tibial plateau offset (%) and the posterior tibial slope (°) of the anterior cruciate ligament (ACL) failure group.

**Figure 5:**
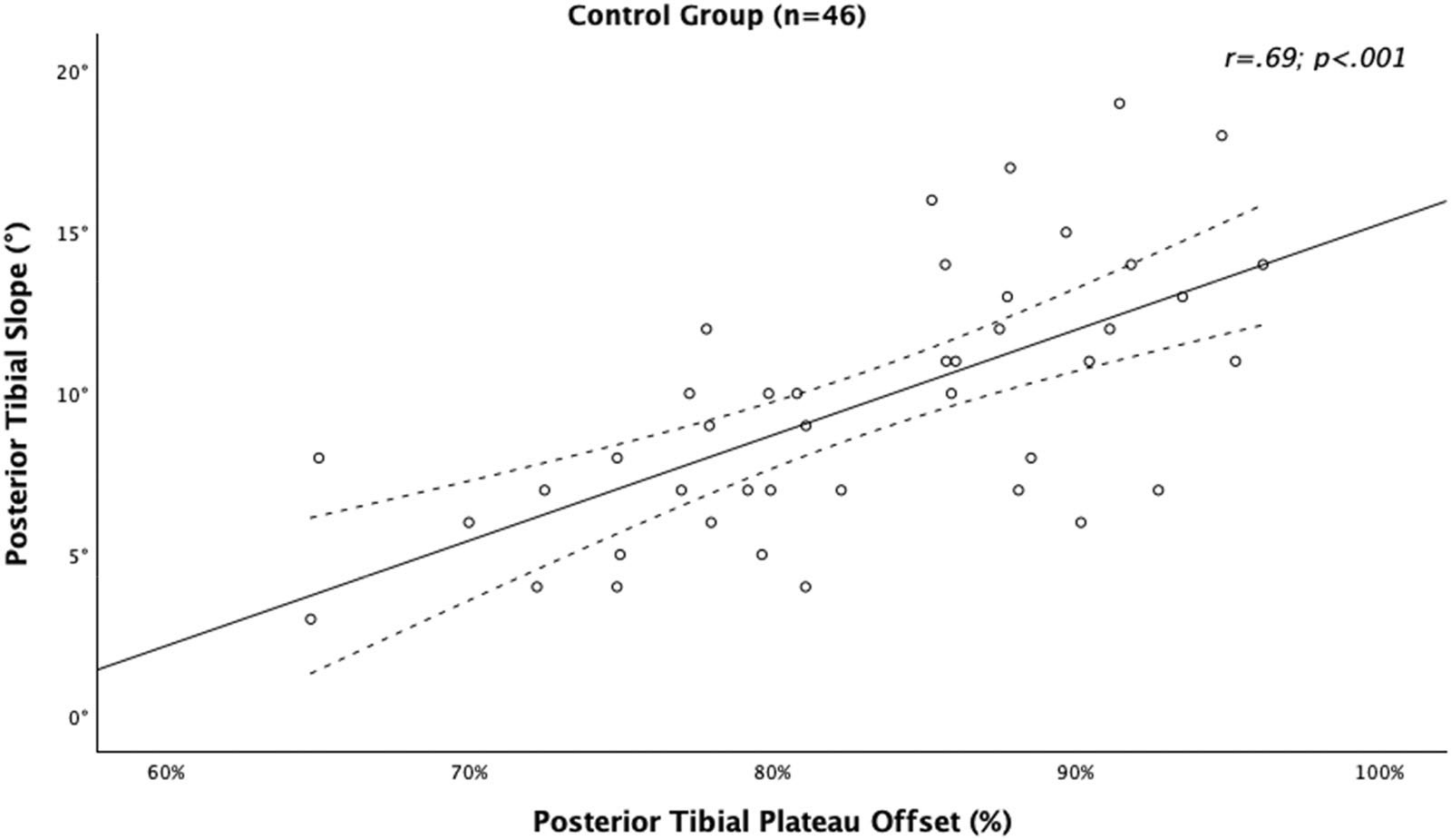
Scatter plot with a trendline (black line) and confidence intervals (dotted lines) illustrating the positive correlation of the posterior tibial plateau offset (%) and the posterior tibial slope (°) of the control group.

A total of 146 (*n* = 103 ACL graft failure, *n* = 43 control) patients were included in this study. Of the study population, 45% were female (*n* = 65) and 60% (*n* = 88) were left knees. The demographic data is visualised in Table 1. The mean posterior tibial offset was 82% ± 7% (65%–99%) and the mean PTS was 12° ± 3 (7–20). In the overall study group (ACL graft failure plus control group), the correlation analysis resulted in a significant correlation between the PTPO and PTS (*r* = 0.49, *p* < 0.001), indicating that higher posterior tibial plateau offset values were associated with higher PTS values (Figure 3). The same effect of a positive correlation of PTS with PTO was detected when separating the groups into ACL graft failure group (Table 2, Figure 4) and control group (Table 3, Figure 5). There was no statistical difference in PTO between the ACL graft failure (83%) and the control group (82%, *p* < 0.05). The interrater and interrater readability between the three raters for PTS and posterior tibial offset ranged from 0.8 to 0.9 (*p* < 0.05) for all measurements.

**Table 1:** Descriptive statistics of the overall study collective (ACL graft insufficient and control).

ACL graft failure + control group (*N* = 146)			
Parameter	Mean ± SD	Min.	Max.
Age (years)	31 ± 8	18	52
Sex (female, *n*)	60 (41%)	n/a	n/a
Laterality (left, *n*,%)	68 (47%)	n/a	n/a
PTS (°)	12 ± 4	3	20
PTO (%)	82 ± 7	65	99
Distance P—A (mm)	50 ± 6	35	72
Distance P—tibial shaft axis (mm)	42 ± 6	26	57

Abbreviations: A, anterior tibial articular margin; BMI, body mass index; mm, millimetres; P, posterior tibial margin; PTO, posterior tibial offset; PTS, posterior tibial slope (°); SD, standard deviation.

**Table 2:** Descriptive statistics of the overall study collective (anterior cruciate ligament [ACL] graft insufficient and control).

ACL graft failure *N* = 103			
Parameter	Mean ± SD	Min.	Max.
Age (years)	30 ± 7.9	18	44
Sex (female, *n*)	39 (49%)	n/a	n/a
Laterality (left, *n*)	46 (45%)	n/a	n/a
PTS (°)	12 ± 3	7	20
PTO (%)	83 ± 6	66	94
Distance P—A (mm)	50 ± 7	39	63
Distance P—tibial shaft axis (mm)	42 ± 5	32	57

Abbreviations: A, anterior tibial articular margin; BMI, body mass index; mm, millimetres; P, posterior tibial margin; PTO, posterior tibial offset; PTS, posterior tibial slope (°); SD, standard deviation.

**Table 3:** Descriptive statistics of the control group.

Control group *N* = 43			
Parameter	Mean ± SD	Min.	Max.
Age (years)	41 ± 16	18	52
Sex (female, *n*,%)	21 (28)	n/a	n/a
Laterality (left, *n*, %)	22 (51%)	n/a	n/a
PTS (°)	10 ± 4	7	18
PTO (%)	82 ± 8	68	99
Distance P—A (mm)	51 ± 7	35	72
Distance P—tibial shaft axis (mm)	42 ± 7	26	56

Abbreviations: A, anterior tibial articular margin; BMI, body mass index; mm, millimetres; P, posterior tibial margin; PTO, posterior tibial offset; PTS, posterior tibial slope (°); SD, standard deviation.

## DISCUSSION

The most significant finding of this study was the observation of a moderate, significant correlation between the PTPO and the PTS, indicating that knees with a higher PTS present with a greater posterior tibial plateau offset, which reflects a more posteriorly oriented tibial plateau. Furthermore, the posterior tibial plateau offset is a simple and repeatable measure of the posterior positioning of the proximal tibial plateau relative to the tibial shaft axis. The interindividual variability of the PTPS was high in patients who experienced an ACL graft failure and in a control group.

The morphology of the proximal tibia has significant biomechanical and clinical relevance, particularly in the context of ACL injuries [3, 14, 20]. Within the sagittal morphology of the tibia, the PTS is the most widely investigated parameter, with higher PTS values consistently reported in cohorts with ACL tears and graft failures [4, 7, 22]. However, PTS only describes the posterior inclination of the tibial plateau and not its posterior positioning relative to the tibial shaft. The present study adds to the current knowledge by quantifying this posterior positioning through the posterior tibial plateau offset, showing a higher PTPO, meaning a more posterior orientation of the tibial plateau in relation to the tibial shaft axis.

Multiple parameters were published to describe the proximal tibial morphology in addition to the PTS [1, 9, 21, 27]. One study investigated metaphyseal inclination and found a correlation between PTS and posterior metaphyseal tilt, showing that increased slope is associated with posteriorly inclined metaphysis [9]. The findings of the current study support the concept that the posterior inclination of the tibial plateau translates into a posterior positioning of the tibial plateau in relation to the tibial shaft. Another study has identified that tibial tubercle overgrowth and tibial tubercle height both positively correlated with PTS [21]. The PTPO does not take the tibial tubercle into account and may be seen as a complementary variable to the tibial tubercle height and angle.

A magnetic resonance imaging (MRI) based study showed that the posterior tibial offset ratio did not identify significant differences in ACL‐injured versus intact knees [27]. Notably, MRI‐based measurements are prone to underestimating the PTS. This occurs due to a more posteriorly tilted tibial shaft axis in the two‐circle method used. The method used to define the proximal tibial anatomical axis in the current study is a widely accepted method and the current gold standard in measuring the PTS. It does not rely on the tibial tubercle and benefits from a long tibial shaft axis, potentially offering a more anatomically accurate assessment [11, 19].

The correlation between posterior tibial offset and PTS may have clinical implications. As previous studies showed, an increased PTS leads to an increased strain on the ACL, ACL‐graft, and the posterior horn of the medial and lateral meniscus [2, 25]. An increased posterior tibial plateau offset may therefore be a factor that influences the load distribution in knee kinematics, especially in the context of anterior closing wedge HTOs. Another clinical implication may occur in osteotomy planning. A high posterior tibial plateau offset may pose challenges for infratubercle anterior closing wedge HTO, as the curvature of the posterior cortex may limit the ability to reach the desired hinge point. In turn, non‐optimal hinge placement may increase the risk of mal‐correction or hinge fractures, as this has been shown in medial opening wedge HTOs [18].

The PTS is currently one of the most discussed risk factors in ACL graft failure. Subjectively, the authors noted that the PTO also showed considerable variability. Therefore, the aim of this study was to quantify the PTO to support one aspect of the hypothesis: that the PTO correlates with the PTS in a cohort of ACL failures, which naturally demonstrate higher PTS values. The study also attempted to determine whether PTO correlates with PTS in a control group. In future studies with larger sample sizes, it could be investigated whether interchangeable or modifying effects exist between PTS and PTO (e.g., whether the risk of ACL graft failure in patients with high PTS is increased or reduced by a high PTO). This could help explain why many patients with high PTS values still have intact ACLRs.

This study has limitations that should be considered when interpreting the findings. First, the analysis was conducted on a specific patient group consisting solely of individuals who had ACL graft failure. Therefore, the PTS of this group was generally higher and may not reflect the general population. In this study, it was aimed to determine if the posterior tibial plateau offset increases with an increase in PTS and to assess the reliability and repeatability of measuring the posterior tibial offset. Second, the measurements were performed using two‐dimensional lateral radiographs, which provide information limited to the sagittal plane. This approach does not account for three‐dimensional variations in tibial morphology. The measurement technique in this study followed the current gold standard for measuring the PTS, and for an appropriate comparability, the measurements for the posterior tibial plateau offset were performed on radiographs too. Finally, the study focused exclusively on morphological parameters and did not assess their functional implications. The correlation between posterior tibial plateau offset and clinical and functional outcomes or biomechanical implications may be further investigated.

The strengths of this study include the implementation of a simple and reliable measurement method to quantify the PTO in clinical practice. This may have important clinical implications, such as for planning an anterior closing wedge HTO. The PTO could influence the angle and length of the osteotomy cuts. Finally, the study highlights the variability of the PTO in both a cohort with naturally higher PTS and a control group, demonstrating that this is not merely a morphological characteristic specific to one population.

## CONCLUSION

The posterior tibial plateau offset is a simple and repeatable measure of the posterior positioning of the tibial plateau related to the tibial shaft axis. A higher PTS is associated with a higher posterior tibial plateau offset and the variability across the patients with ACL graft failure and the control group was high. When planning osteotomy levels in the highly sloped proximal tibia, surgeons may want to consider high offset variability to optimise individualised planning for patients with failed ACL reconstruction.

## AUTHOR CONTRIBUTIONS

**Romed P. Vieider**: Conceptualisation; methodology; writing—original draft preparation; writing—review and editing. **Robert E. Bilodeau**: Methodology; writing—original draft preparation; writing—review and editing. **Mahmut E. Kayaalp**: Methodology; writing—review and editing. **Tyler M. Hauer**: Conceptualisation; methodology; writing—review and editing. **Karina Dias**: Writing—review and editing. **Ting Cong**: Writing—review and editing. **Jonathan D. Hughes**: Writing—review and editing. **Volker Musahl**: Conceptualisation; writing—review and editing; supervision.

## CONFLICT OF INTEREST STATEMENT

Volker Musahl declares educational grants, consulting fees and speaking fees from Smith & Nephew plc, educational grants from Arthrex and DePuy/Synthes, is a board member of the International Society of Arthroscopy, Knee Surgery and Orthopaedic Sports Medicine (ISAKOS), and deputy editor‐in‐chief of Knee Surgery, Sports Traumatology, Arthroscopy (KSSTA). Jonathan D. Huhges: Associate Editor of Knee Surgery, Sports Traumatology, Arthroscopy (KSSTA), paid consultant to Smith and Nephew, editorial board of Annals of Joint. Mahmut E. Kayaalp: Associate Editor of Knee Surgery, Sports Traumatology, Arthroscopy (KSSTA), Member of the ESSKA U‐45 Scientific Committee. The remaining authors declare no conflicts of interest.

## ETHICS STATEMENT

Institutional Review Board approval was obtained by the University of Pittsburgh prior to the initiation of the study (STUDY19030196).

## Data Availability

The data that support the findings of this study are available on request from the corresponding author. The data are not publicly available due to privacy or ethical restrictions.

## References

[1] Akti S , Akti S , Zeybek H , Celebi NO , Karaguven D , Cankaya D . Anterior metaphyseal angle; much less individual variation in determining the posterior slope of the tibia. J Orthop Sci. 2023;28:1046–1051. 10.1016/j.jos.2022.06.017 35864026

[2] Amirtharaj MJ , Pourmodheji R , Wheatley MGA , Leluc J , Pechstein AE , Hirth JM , et al. Sagittal slope‐reducing high tibial osteotomy decreases anterior cruciate ligament force and coupled internal tibial rotation under pivoting loads: a computational modeling study. Am J Sports Med. 2025;53:1614–1621. 10.1177/03635465251334649 40292779

[3] Bayer S , Meredith SJ , Wilson KW , De Sa D , Pauyo T , Byrne K , et al. Knee morphological risk factors for anterior cruciate ligament injury: a systematic review. J Bone Jt Surg. 2020;102:703–718. 10.2106/JBJS.19.00535 31977822

[4] Beel W , Schuster P , Michalski S , Mayer P , Schlumberger M , Hielscher L , et al. High prevalence of increased posterior tibial slope in ACL revision surgery demands a patient‐specific approach. Knee Surg Sports Traumatol Arthrosc. 2023;31:2974–2982. 10.1007/s00167-023-07313-2 36622421

[5] Brandon ML , Haynes PT , Bonamo JR , Flynn MI , Barrett GR , Sherman MF . The association between posterior‐inferior tibial slope and anterior cruciate ligament insufficiency. Arthrosc J Arthrosc Rel Surg. 2006;22:894–899. 10.1016/j.arthro.2006.04.098 16904590

[6] Chang TW , Huang CH , McClean CJ , Lai YS , Lu YC , Cheng CK . Morphometrical measurement of resected surface of medial and lateral proximal tibia for Chinese population. Knee Surg Sports Traumatol Arthrosc. 2012;20:1730–1735. 10.1007/s00167-011-1749-9 22048749

[7] Dejour DH , Dan MJ , Cance N . Editorial commentary: posterior tibial slope‐reducing osteotomy should be considered in patients having primary anterior cruciate ligament reconstruction if posterior tibial slope is greater than 12° to 14°. Arthrosc J Arthrosc Rel Sur. 2025;41:3196–3199. 10.1016/j.arthro.2025.03.004 40081626

[8] Dejour H , Bonnin M . Tibial translation after anterior cruciate ligament rupture. Two radiological tests compared. J Bone Jt Surg Br. 1994;76:745–749. 8083263

[9] Demey G , Giovannetti de Sanctis E , Mesnard G , Müller JH , Saffarini M , Dejour DH . Posterior tibial slope correlated with metaphyseal inclination more than metaphyseal height. Knee. 2023;44:262–269. 10.1016/j.knee.2023.08.007 37717277

[10] Faschingbauer M , Sgroi M , Juchems M , Reichel H , Kappe T . Can the tibial slope be measured on lateral knee radiographs? Knee Surg Sports Traumatol Arthrosc. 2014;22:3163–3167. 10.1007/s00167-014-2864-1 24482216

[11] Gwinner C , Fuchs M , Sentuerk U , Perka CF , Walter TC , Schatka I , et al. Assessment of the tibial slope is highly dependent on the type and accuracy of the preceding acquisition. Arch Orthop Trauma Surg. 2019;139:1691–1697. 10.1007/s00402-019-03201-y 31104087

[12] Hashemi J , Chandrashekar N , Gill B , Beynnon BD , Slauterbeck JR , Schutt, Jr. RC , et al. The geometry of the tibial plateau and its influence on the biomechanics of the tibiofemoral joint. J Bone Jt Surg Am Vol. 2008;90:2724–2734. 10.2106/JBJS.G.01358 PMC2663332 19047719

[13] Kabirian N , Jiang D , Fleming CME , Marecek GS . Restoring condylar width: radiographic relationship between the lateral tibial plateau and lateral femoral condyle in normal adult knees. J Orthop Trauma. 2019;33:180–184. 10.1097/BOT.0000000000001412 30893217

[14] Kayaalp ME , Winkler P , Zsidai B , Lucidi GA , Runer A , Lott A , et al. Slope osteotomies in the setting of anterior cruciate ligament deficiency. J Bone Jt Surg. 2024;106:1615–1628. 10.2106/JBJS.23.01352 39066689

[15] Kim Y , Onishi S , Kubota M , Khakha R , Ishijima M , Ollivier M . In proximal tibial anterior closing wedge (slope changing) osteotomy lower starting points imply larger bone resection. Orthop Traumatol Surg Res. 2025;111:103979. 10.1016/j.otsr.2024.103979 39197639

[16] Kohn MD , Sassoon AA , Fernando ND . Classifications in brief: Kellgren‐Lawrence classification of osteoarthritis. Clin Orthop Rel Res. 2016;474:1886–1893. 10.1007/s11999-016-4732-4 PMC4925407 26872913

[17] Lott A , James MG , Kaarre J , Höger S , Kayaalp ME , Ollivier M , et al. Around‐the‐knee osteotomies part II: surgical indications, techniques and outcomes—state of the art. J ISAKOS. 2024;9:658–671. 10.1016/j.jisako.2024.04.002 38604568

[18] Moon DK , Seo MS , Kim CW , Cho SH , Nam DC , Byun JH , et al. The influence of different hinge position on PTS during HTO: comparison between open‐wedge and closed‐wedge HTO. Euro J Orthop Surg Traumatol. 2023;33:1341–1347. 10.1007/s00590-022-03280-5 PMC10126045 35639172

[19] Naendrup JH , Drouven SF , Shaikh HS , Jaecker V , Offerhaus C , Shafizadeh ST , et al. High variability of tibial slope measurement methods in daily clinical practice: comparisons between measurements on lateral radiograph, magnetic resonance imaging, and computed tomography. Knee. 2020;27:923–929. 10.1016/j.knee.2020.01.013 32061503

[20] Nazzal EM , Zsidai B , Pujol O , Kaarre J , Curley AJ , Musahl V . Considerations of the posterior tibial slope in anterior cruciate ligament reconstruction: a scoping review. Curr Rev Musculoskelet Med. 2022;15:291–299. 10.1007/s12178-022-09767-2 PMC9276900 35653051

[21] Rosenthal RM , Hunter CDR , Froerer DL , Featherall J , Metz AK , Ernat JJ , et al. Correlation of tibial tubercle overgrowth with increased posterior tibial slope: a novel radiographic assessment. Orthop J Sports Med. 2024;12:23259671231225660. 10.1177/23259671231225660 PMC10836143 38313754

[22] Salmon LJ , Heath E , Akrawi H , Roe JP , Linklater J , Pinczewski LA . 20‐year outcomes of anterior cruciate ligament reconstruction with hamstring tendon autograft the catastrophic effect of age and posterior tibial slope. Am J Sports Med. 2018;46:531–543. 10.1177/0363546517741497 29244525

[23] Utzschneider S , Goettinger M , Weber P , Horng A , Glaser C , Jansson V , et al. Development and validation of a new method for the radiologic measurement of the tibial slope. Knee Surg Sports Traumatol Arthrosc. 2011;19:1643–1648. 10.1007/s00167-011-1414-3 21298254

[24] Vieider RP , Mehl J , Rab P , Brunner M , Schulz P , Rupp MC , et al. Malrotated lateral knee radiographs do not allow for a proper assessment of medial or lateral posterior tibial slope. Knee Surg Sports Traumatol Arthrosc. 2024;32:1462–1469. 10.1002/ksa.12170 38629758

[25] Winkler PW , Chan CK , Polamalu SK , Lucidi GA , Wagala NN , Hughes JD , et al. Meniscal forces and knee kinematics are affected by tibial slope modifying high tibial osteotomy. Knee Surg Sports Traumatol Arthrosc. 2025;33:2345–2355. 10.1002/ksa.12577 PMC12205423 39756014

[26] Winkler PW , Wagala NN , Hughes JD , Lesniak BP , Musahl V . A high tibial slope, allograft use, and poor patient‐reported outcome scores are associated with multiple ACL graft failures. Knee Surg Sports Traumatol Arthrosc. 2022;30:139–148. 10.1007/s00167-021-06460-8 PMC8800919 33517476

[27] Yaka H , Özer M , Kanatli U . Modifiers of the posterior tibial slope as a predisposing factor for anterior cruciate ligament ruptures. Orthop J Sports Med. 2025;13:23259671251337482. 10.1177/23259671251337482 PMC12081971 40386648

